# Positioning double-balloon enteroscopy in the diagnostic algorithm for suspected small bowel Crohn’s disease: a retrospective analysis of decision-making at a tertiary referral centre

**DOI:** 10.3389/fgstr.2026.1699553

**Published:** 2026-04-01

**Authors:** Thomas Sheehan, Cormac Hegarty, Roisin Connaughton, Barry Hall, Orlaith B. Kelly

**Affiliations:** 1 Gastroenterology Department, Connolly Hospital, Dublin, Ireland; 2 Royal College of Surgeons in Ireland, Dublin, Ireland

**Keywords:** capsule endoscopy, Crohn’s disease, diagnostic yield, double-balloon enteroscopy, small bowel

## Abstract

**Background:**

Small bowel capsule endoscopy (SBCE) enables non-invasive mucosal assessment of the small bowel, while double-balloon enteroscopy (DBE) allows histological confirmation and therapeutic intervention. Appropriate patient selection is essential to maximise diagnostic yield and minimise unnecessary invasive procedures.

**Methods:**

We performed a retrospective analysis of patients referred for investigation of suspected small bowel Crohn’s disease over a two-year period at a tertiary referral centre. Demographic data, prior investigations, SBCE findings, and subsequent DBE decisions were recorded. The primary outcome was the decision to proceed to DBE. Secondary outcomes included the diagnostic yields of SBCE and DBE. Multivariate logistic regression was used to identify factors associated with DBE referral.

**Results:**

Ninety-eight patients with complete data were included. SBCE was performed as the initial investigation in 90.8%, while 5.5% proceeded directly to DBE for therapeutic or histological indications. The SBCE-to-DBE conversion rate was 30.4%. SBCE alone established or excluded inflammatory bowel disease in 70% of patients. Among those undergoing DBE, Crohn’s disease was confirmed in 21% and excluded in 79%. Increasing age (OR 1.04 per year; 95% CI 1.01–1.07) and diagnostic uncertainty on SBCE (OR 2.0; 95% CI 1.8–3.5) independently predicted DBE referral.

**Conclusion:**

SBCE is diagnostic in the majority of patients with suspected small bowel Crohn’s disease and functions effectively as a triage tool. DBE should be reserved for cases requiring histological confirmation, clarification of indeterminate findings, assessment of proximal disease, or therapeutic intervention.

## Introduction

Small bowel Crohn’s disease (CD) remains diagnostically and therapeutically challenging, particularly in healthcare settings with limited access to advanced small bowel investigations beyond routine ileocolonoscopy and cross-sectional imaging. Isolated mucosal involvement of the small bowel may be missed using conventional diagnostic approaches, leading to delayed diagnosis and treatment initiation.

Small bowel capsule endoscopy (SBCE) is a safe, non-invasive modality that enables complete visualisation of the small bowel mucosa. When appropriately indicated, SBCE demonstrates a high diagnostic yield in suspected small bowel CD (1, 2). However, it has important limitations, including the inability to obtain tissue for histological confirmation or perform therapeutic interventions. In addition, there is a recognised risk of capsule retention, particularly in patients with established or suspected Crohn’s disease and underlying stricturing pathology, with reported retention rates ranging from 0–5.4% in suspected CD and higher rates in established disease (1, 3, 4).

Double-balloon enteroscopy (DBE) may be used as an adjunctive investigation, as recommended by the American College of Gastroenterology, allowing for direct mucosal assessment, histological sampling, and therapeutic interventions such as stricture dilatation or capsule retrieval (1, 5). DBE utilises an overtube-assisted system to achieve deep small bowel intubation via antegrade or retrograde approaches. Reported diagnostic yields for DBE in suspected small bowel CD range from 60–87%, with a substantial proportion of patients experiencing changes in clinical management following the procedure (1, 5–7). Historically, obtaining diagnostic histology from the small bowel—particularly in duodenal or proximal disease—posed a significant challenge prior to the availability of deep enteroscopy techniques.

The aim of this study was to assess the diagnostic yield of SBCE and DBE in patients with suspected new small bowel Crohn’s disease at a tertiary referral centre, and to evaluate the clinical and investigational factors associated with the decision to proceed to DBE.

## Methods

### Study design and patient selection

A retrospective cohort analysis was performed including all patients referred to a tertiary SBCE service for suspected small bowel Crohn’s disease over a two-year period. Patients were identified from a prospectively maintained local capsule endoscopy database.

Inclusion criteria comprised patients referred for suspected small bowel CD who underwent a completed SBCE examination. Patients with incomplete capsule studies or unavailable reports were excluded.

### Data collection

Patient demographics and clinical data were extracted from electronic medical records and endoscopy reporting systems. Variables recorded included referral indication and presenting symptoms, prior ileocolonoscopy and histology results, faecal calprotectin levels, and cross-sectional imaging findings, including magnetic resonance enterography (MRE).

SBCE and DBE reports, as well as outcomes from multidisciplinary team discussions, were reviewed. All data were anonymised and stored in a coded, password-protected database.

### Capsule endoscopy and enteroscopy technique

Only PillCam™ SB3 capsule examinations were included for consistency. All SBCE studies were interpreted by a single expert consultant gastroenterologist with subspecialty expertise in capsule endoscopy.

DBE procedures were performed according to standard institutional protocols. Indications included diagnostic clarification following SBCE, histological confirmation, or therapeutic intervention.

### Outcomes

The primary outcome was the decision to proceed to diagnostic DBE following referral or SBCE. Secondary outcomes included the diagnostic yield of SBCE and DBE for inflammatory bowel disease.

### Statistical analysis

Descriptive statistics were reported as medians with interquartile ranges (IQRs) for continuous variables and frequencies with percentages for categorical variables. Comparisons between DBE and non-DBE groups were performed using the Mann–Whitney U test. Associations between categorical variables were assessed using chi-square analysis.

Variables demonstrating significant associations on univariate analysis were entered into a binary logistic regression model. Statistical analyses were performed using IBM SPSS Statistics. A two-sided p value <0.05 was considered statistically significant.

## Results

### Study cohort

A total of 115 SBCE referrals for suspected small bowel inflammatory bowel disease were reviewed. Six patients proceeded directly to DBE due to anticipated therapeutic need (strictures, n=4; active bleeding, n=2). Eleven patients were excluded due to incomplete data. The remaining 98 patients comprised the final analytic cohort.

### Patient demographics and referral indications

The median age was 40.5 years, and 48% were female. Primary referral symptoms included diarrhoea (47%), abdominal pain (23%), anaemia or gastrointestinal bleeding (12%), weight loss (8%), and other symptoms (10%). Over 92% of patients had completed pre-referral laboratory investigations and upper and lower gastrointestinal endoscopy.

**Figure 1:**
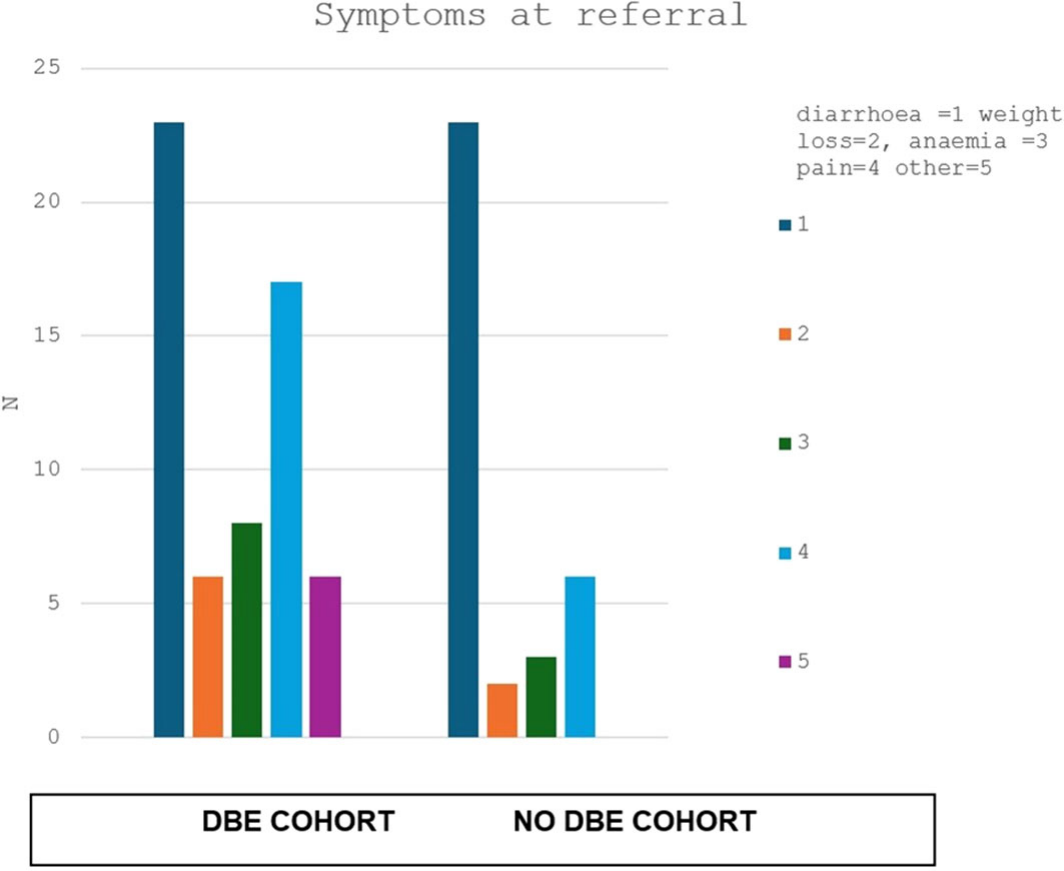
Symptoms profile at time of referral.

Cross-sectional imaging was available in 28 patients (28%), with findings suggestive of enteritis reported in eight cases (33%) (**Table 1** and **Figure 1**).

**Table 1:** Patient demographics.

Patient Demographic	Value
Age (Median, years)	40.5
Gender (M/F %)	52% / 48%
Symptoms at Referral (%)	47% diarrhoea, 8% weight loss, 12% anaemia/bleeding, 23% pain, 10% other
Colonoscopy Performed (%)	95%

### Diagnostic pathway and findings

SBCE was the initial investigation in 90.8% of patients. The capsule-to-DBE conversion rate was 30.4%. SBCE alone established a diagnosis of inflammatory bowel disease in 37% of cases and confidently excluded IBD in 33%.

Among patients undergoing DBE, Crohn’s disease was confirmed in six cases (21%), while suspected IBD was excluded in 22 cases (79%). The most common indication for DBE was diagnostic uncertainty following SBCE, particularly in the presence of non-specific enteritis.

### Factors associated with DBE referral

Increasing age was associated with DBE referral (median 54 vs 34 years; p<0.003). Gender was not associated with DBE use. Presenting symptoms of persistent diarrhoea or blood loss were significantly associated with DBE referral (χ²=8.105; p=0.0439).

On multivariate analysis, increasing age (OR 1.04 per year; 95% CI 1.01–1.07; p=0.0053) and diagnostic uncertainty on SBCE (OR 2.0; 95% CI 1.8–3.5; p<0.001) independently predicted DBE referral.

## Discussion

### Small bowel capsule endoscopy in Crohn’s disease

Small bowel capsule endoscopy (SBCE) has become a cornerstone investigation in the evaluation of suspected small bowel Crohn’s disease due to its ability to provide high-resolution, pan-enteric visualisation of mucosal pathology. Its sensitivity for early inflammatory lesions, including aphthous ulceration and subtle mucosal breaks, exceeds that of cross-sectional imaging, making it particularly valuable in patients with negative ileocolonoscopy but ongoing clinical suspicion (1, 2). The diagnostic accuracy of SBCE for small bowel Crohn’s disease is 71.5%, with sensitivity of 89.6% and specificity of 86.2%, superior to both MR enterography (67.9% accuracy) and CT enterography (52.3% accuracy) (2). Previous studies have demonstrated a high negative predictive value for SBCE of 96%, supporting its role in confidently excluding small bowel Crohn’s disease when findings are normal (1, 3, 8).

In our cohort, SBCE alone established or refuted a diagnosis of inflammatory bowel disease in the majority of patients, reinforcing its effectiveness as a first-line investigation. However, SBCE findings must be interpreted within clinical context, as non-specific inflammatory changes are not pathognomonic for Crohn’s disease. The absence of consensus regarding which capsule endoscopy findings constitute a definitive diagnosis of Crohn’s disease remains a fundamental limitation (1, 3). While the Lewis score quantifies small bowel inflammatory burden based on villous appearance, ulcers, and strictures, it does not distinguish Crohn’s disease from other inflammatory conditions (1). Specific features that favour Crohn’s disease include cobblestone appearance, longitudinal or irregular ulcers, and circumferential or longitudinal alignment of diminutive lesions, particularly in the proximal small bowel (3, 9, 10). However, the inability to obtain histological confirmation remains problematic, particularly in cases of indeterminate enteritis, isolated erosions, or lymphoid nodularity. These diagnostic grey zones were reflected in our data, where non-specific enteritis on SBCE was the strongest driver for escalation to DBE. Thus, while SBCE serves as an excellent screening and triage tool, it cannot be considered definitive in all cases.

### Role of cross-sectional imaging

Cross-sectional imaging, particularly MR enterography (MRE), plays an essential complementary role in the diagnostic assessment of suspected small bowel Crohn’s disease. Unlike SBCE, MRE allows assessment of transmural inflammation, bowel wall thickness, stricturing, fistulising disease, and extraluminal complications (1, 3). These features are critical for disease phenotyping according to the Montreal classification, risk stratification, and procedural planning, particularly when therapeutic intervention or capsule retention is a concern (3, 11). MRE features such as wall enhancement, mucosal lesions, and T2 hyperintensity are suggestive of intestinal inflammation, and improvement in these parameters correlates with better clinical outcomes regarding hospitalisation, surgery, and corticosteroid use (1, 3).

However, MRE has recognised limitations in detecting early or purely mucosal disease, where inflammatory changes may fall below the threshold of radiologic detection (3, 12). SBCE demonstrates superior diagnostic yield for superficial and proximal small bowel lesions, with one study showing SBCE detected jejunal inflammation in 31.9% of patients compared to 6.4% with MRE (3). Consequently, MRE and SBCE should be viewed as synergistic rather than competing modalities. In clinical practice, MRE often informs the safety and appropriateness of capsule endoscopy by identifying strictures that increase retention risk, while SBCE refines mucosal assessment when imaging findings are equivocal or negative despite ongoing symptoms (1, 12).

In our cohort, the relatively low utilisation of MRE reflects real-world constraints including access limitations and waiting times rather than diminished clinical value. Importantly, when performed, imaging findings frequently guided subsequent investigation strategy, particularly in identifying stricturing or penetrating disease that necessitated DBE for therapeutic or histologic purposes. These observations support the integration of MRE into a structured diagnostic pathway rather than its use as an isolated confirmatory test. Given the need for sequential imaging in young patients, those with upper gastrointestinal disease, penetrating disease, or those requiring steroids, biologics, and surgery, MRE is preferred over CTE to avoid cumulative radiation exposure (1, 3).

### Double-balloon enteroscopy

Double-balloon enteroscopy (DBE) represents the most invasive but also the most diagnostically definitive modality in the evaluation of small bowel disease. Its principal advantage lies in the ability to obtain targeted biopsies, perform therapeutic interventions such as stricture dilation or haemostasis, and directly assess lesions identified on prior investigations (3). The American College of Gastroenterology states that deep enteroscopy is not part of routine diagnostic testing in patients with suspected Crohn’s disease, but may provide additional information in patients who require biopsy/sampling of small bowel tissue to make a diagnosis (3). DBE has a diagnostic yield as high as 80% in patients with suspected Crohn’s disease and is more sensitive than multiple radiographic imaging techniques in detecting lesions (5).

In our study, DBE was most frequently pursued following SBCE when findings were indeterminate or non-specific rather than overtly diagnostic. Notably, DBE more often excluded Crohn’s disease than confirmed it, highlighting its role in refining diagnosis rather than simply increasing diagnostic yield. This finding is consistent with published data showing that in patients referred for suspected Crohn’s disease, DBE confirmed the diagnosis in only 40% of cases, with 63% of patients previously diagnosed with Crohn’s disease at outside institutions having the diagnosis confirmed on DBE (6). This is particularly relevant in older patients, where the differential diagnosis is broader and the consequences of misdiagnosis are significant. The association between increasing age and DBE referral observed in our cohort likely reflects heightened clinical concern for alternative pathologies that require tissue confirmation.

DBE impacts management in 77-82% of patients with suspected or known Crohn’s disease (1, 6). However, it should not be regarded as a routine second-line investigation following SBCE. Rather, its use should be guided by a clear diagnostic or therapeutic question, including the need for histology, assessment of proximal small bowel disease, or intervention for complications such as stricture dilation or capsule retrieval (3, 6, 13). Though when deemed necessary, DBE intervention should be sought after, as recommended by a 75% target in ESGE guidelines. This targeted approach maximises diagnostic utility while minimising procedural risk, which includes perforation in approximately 1% of cases and failure to reach target lesions in up to 17% of procedures (1, 6, 11).

### Crohn’s disease mimics and the role of histology

A critical limitation of both SBCE and cross-sectional imaging is their inability to reliably distinguish Crohn’s disease from other causes of small bowel inflammation without histological correlation. A range of conditions—including small bowel lymphoma, NSAID-induced enteropathy, infectious enteritis (including tuberculosis), ischaemia, vasculitis (including Behçet’s disease), endometriosis, and drug reactions—can produce endoscopic and radiologic findings that overlap with Crohn’s disease [14–19]. This diagnostic overlap is particularly relevant in patients with atypical presentations, isolated small bowel involvement, or late-onset disease.

Histological examination remains the cornerstone of diagnosis, characterised by transmural inflammation with architectural distortion, lymphoid infiltrates, and epithelioid granulomas (though granulomas are seen in fewer than 20% of biopsies) [14, 19]. DBE uniquely addresses this diagnostic gap by enabling tissue acquisition from targeted lesions, allowing for definitive exclusion of Crohn’s mimics. In our cohort, the high proportion of DBE procedures that ultimately refuted a diagnosis of Crohn’s disease underscores the importance of histological confirmation in selected patients. This finding reinforces that DBE adds value not by increasing sensitivity alone, but by improving diagnostic specificity and preventing inappropriate long-term immunosuppressive therapy.

DBE is also especially relevant in suspected proximal or duodenal Crohn’s disease, where lesions may lie beyond the reach of standard oesophagogastroduodenoscopy yet remain poorly characterised by capsule endoscopy alone. In such cases, histological confirmation is essential to distinguish Crohn’s disease from peptic, medication-related, or other inflammatory conditions affecting the upper small bowel. Accurate diagnosis is crucial, as once a diagnosis of inflammatory bowel disease has been established, it is very difficult to “undiagnose” the condition when an alternative diagnosis or mimic has been subsequently identified [16].

### Proposed diagnostic algorithm

**Figure 2:**
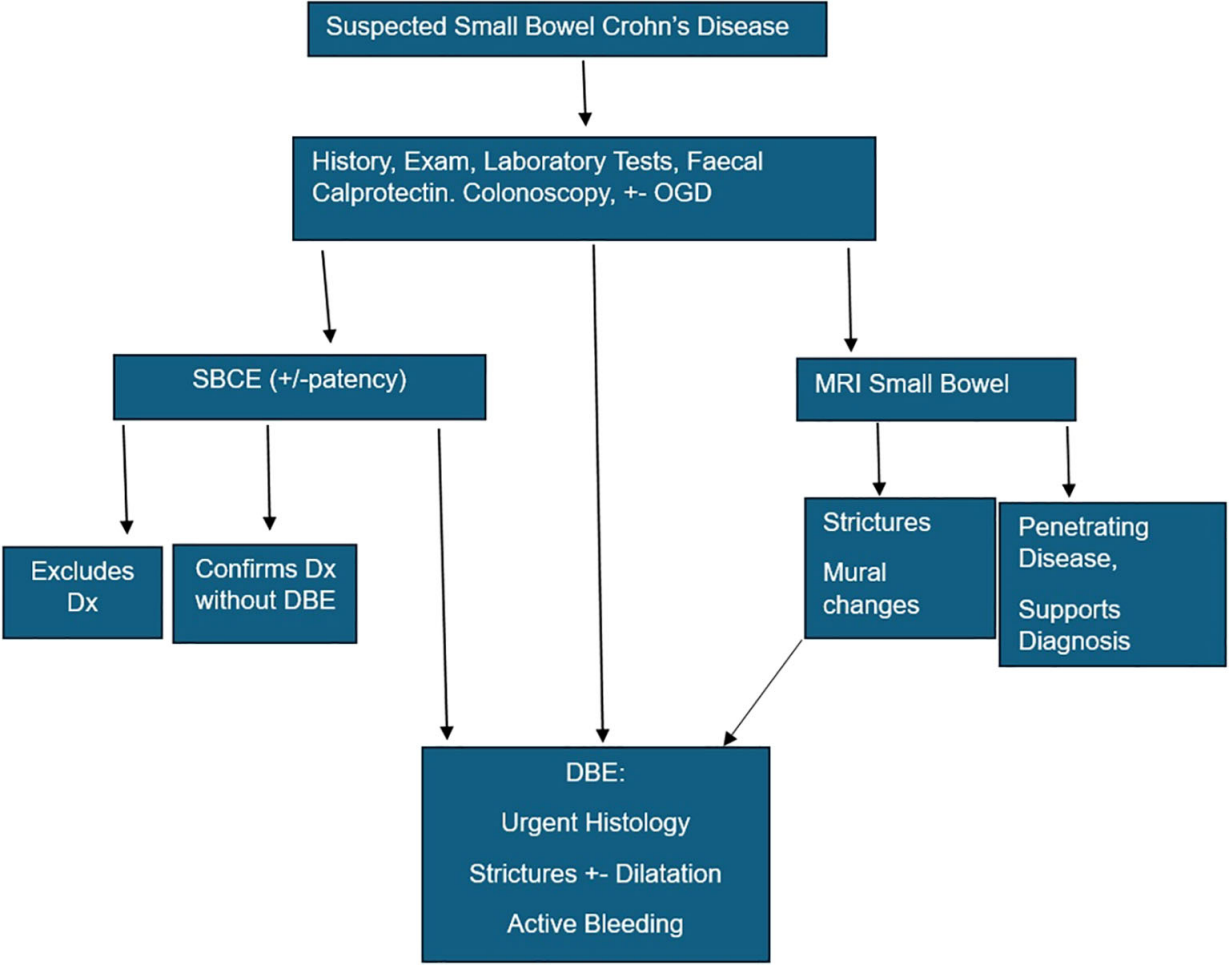
Proposed diagnostic algorithm for SBCD.

Informed by these findings and current evidence, we propose a structured, phenotype-driven diagnostic algorithm for suspected small bowel Crohn’s disease (**Figure 2**), which integrates SBCE, cross-sectional imaging, and DBE according to the specific clinical question being addressed. In this framework, SBCE functions as the primary investigation for sensitive mucosal assessment and is sufficient to confirm or exclude Crohn’s disease in the majority of patients, given its 96% negative predictive value and superior diagnostic accuracy compared to cross-sectional imaging (1, 2, 4). Cross-sectional imaging, particularly MR enterography, plays a complementary role by defining disease phenotype, identifying transmural inflammation, stricturing, or penetrating complications, and informing procedural risk, particularly capsule retention (1, 3, 9).

DBE is positioned as a targeted problem-solving investigation rather than a routine second-line test, reserved for cases in which: (1) histological confirmation is required to exclude mimics of Crohn’s disease; (2) indeterminate or non-specific SBCE findings persist despite clinical suspicion; (3) proximal or duodenal disease is suspected; or (4) therapeutic intervention such as stricture dilation or capsule retrieval is indicated (3, 10–12). This approach reflects the unique ability of DBE to obtain tissue diagnosis and exclude mimics of Crohn’s disease that cannot be reliably distinguished by capsule endoscopy or imaging alone. By aligning investigation choice with diagnostic uncertainty and disease phenotype, this algorithm supports more efficient, clinically grounded use of advanced small bowel endoscopy and may reduce unnecessary invasive procedures while preserving diagnostic accuracy and preventing misdiagnosis that could lead to inappropriate immunosuppressive therapy.

## Data Availability

The raw data supporting the conclusions of this article will be made available by the authors, without undue reservation.
